# Application of microvascular ultrasound-assisted thyroid imaging report and data system in thyroid nodule risk stratification

**DOI:** 10.1186/s13244-024-01806-5

**Published:** 2024-09-23

**Authors:** Guangrong Ma, Libin Chen, Yong Wang, Zhiyan Luo, Yiqing Zeng, Xue Wang, Zhan Shi, Tao Zhang, Yurong Hong, Pintong Huang

**Affiliations:** 1 Department of Ultrasound in Medicine, The Second Affiliated Hospital of Zhejiang University School of Medicine, Hangzhou, P.R. China; 2 Research Center of Ultrasound in Medicine and Biomedical Engineering, The Second Affiliated Hospital of Zhejiang University School of Medicine, Zhejiang University, Hangzhou, P.R. China; 3 Department of Ultrasound in Medicine, The First Affiliated Hospital of Ningbo University, Ningbo, P.R. China; 4 Department of Thyroid Surgery, The Second Affiliated Hospital of Zhejiang University School of Medicine, Zhejiang University, Hangzhou, P.R. China; 5 Research Center for Life Science and Human Health, Binjiang Institute of Zhejiang University, Hangzhou, P.R. China

**Keywords:** Thyroid, Thyroid cancer, Ultrasound imaging

## Abstract

**Objectives:**

To establish superb microvascular imaging (SMI) based thyroid imaging reporting and data system (SMI TI-RADS) for risk stratification of malignancy in thyroid nodules.

**Methods:**

In total, 471 patients, comprising 643 thyroid nodules, who received conventional ultrasound (US), SMI, and a final diagnosis were extensively analyzed. A qualitative assessment of US features of the nodules was performed followed by univariable and multivariable logistic regression analyses, leading to the construction of the SMI TI-RADS, which was further verified using internal and external validation cohorts.

**Results:**

Among the stand-alone US, predictive factors were the shape and margins of the nodules, echogenicity and echogenic foci, vascularity, extrathyroidal extension, ring-SMI patterns, penetrating vascularity, flow-signal enlarged, and vascularity area ratio. SMI TI-RADS depicted an enhanced area under the receiver operating characteristic curve (AUC) of 0.94 (95% CI: 0.92, 0.96; *p* < 0.001 relative to other stratification systems), a 79% biopsy yield of malignancy (BYM, 189/240 nodules), and a 21% unnecessary biopsy rate (UBR, 51/240 nodules). In the verification cohorts, we demonstrated AUCs, malignancy biopsy yields, and unnecessary biopsy rates of 0.88 (95% CI: 0.83, 0.94), 79% (59/75 nodules), and 21% (16/75 nodules) for the internal cohort, respectively, and 0.91 (95% CI: 0.85, 0.96), 72% (31/43 nodules), and 28% (12/43 nodules) for the external cohort, respectively.

**Conclusion:**

SMI TI-RADS was found to be superior in diagnostic sensitivity, specificity, and efficiency than existing TI-RADSs, showing better stratification of the malignancy risk, and thus decreasing the rate of unnecessary needle biopsy.

**Critical relevance statement:**

To develop an imaging and data system based on conventional US and SMI features for stratifying the malignancy risk in thyroid nodules.

**Key Points:**

- SMI features could improve thyroid nodule risk stratification.
- SMI TI-RADS showed superior diagnostic efficiency and accuracy for biopsy guidance.
- SMI TI-RADS can provide better guidance for clinical diagnosis and treatment of thyroid nodules.

**Graphical Abstract:**

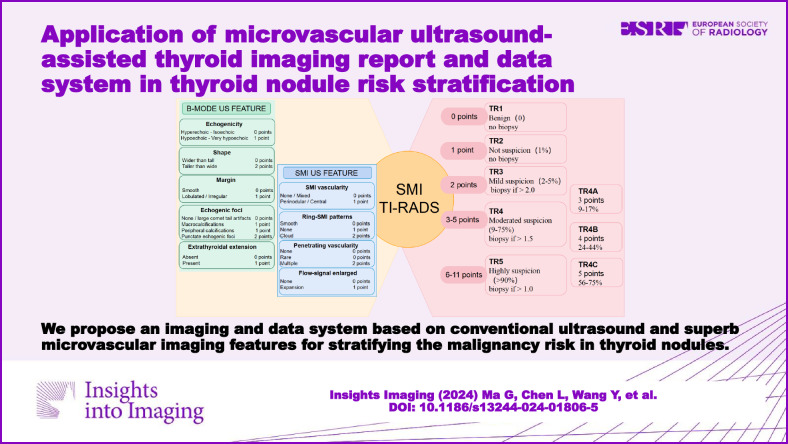

## Introduction

The incidence of thyroid cancer is increasing [1–3]. Ultrasound (US) has shown that between 19% and 68% of the overall population possess thyroid nodules, the majority of which are benign and clinically insignificant, with only 7–15% showing evidence of malignancy [4, 5]. Small malignant tumors usually have a slow course and usually do not cause significant complications or death during the patient’s lifetime, so their detection may constitute overdiagnosis [6, 7]. Fine-needle aspiration (FNA) is both cost-effective and accurate for thyroid nodule evaluation; however, many nodules require neither biopsy nor surgery [4, 8–10]. Thus, stratification of the risk of malignancy is desirable, and the use of risk stratification systems (RSSs) based on the US have been developed for the accurate assessment of the malignancy risk in thyroid modules and thus reduce unnecessary biopsies.

There are currently no fewer than 20 RSSs in use. These include pattern- and non-pattern-based systems, based on the combination of nodule size and conventional US features to provide guidance on the need for FNA biopsy [11–15]. However, most of these RSS do not analyze the vascular distribution and blood flow, both of which are closely related to the biological characteristics of tumors, considering the limitations of vascular characteristic imaging modalities [16, 17]. Examples of these limitations include the technical limitations of color Doppler flow imaging (CDFI) in the detection of small vessels and reduced blood flow due to its imaging principles, and limitations in the application of contrast-enhanced ultrasound imaging in terms of the contrast agent injection and economic conditions [18, 19].

Microvascular ultrasound (MVUS), is a form of Doppler imaging mode that can visualize low-speed blood flow and thus provide a more detailed analysis of vascular blood flow than CDFI, leading to the acquisition of high-quality images of blood flow without the use of contrast agents [20–22]. A meta-analysis found that the sensitivity, specificity, and positive and negative likelihood ratios of SMI (superb microvascular imaging, MVUS based on Canon Medical System) for diagnosing malignant thyroid nodules were 0.84, 0.86, 6.2, and 0.18, respectively, which were all better than CDFI [20, 23].

Here, we propose to use conventional US and MVUS (based on Canon Medical System) to create an easily generalizable and practical risk stratification system for thyroid nodule malignancy, termed SMI thyroid imaging reporting and data system (TI-RADS).

## Materials and methods

### Patient population and data collection

This research received ethical approval from the Second Affiliated Hospital of Zhejiang University School of Medicine (no. 2023-0944), and the participant informed consent requirement was waived as the study was retrospective. Regardless, all subjects who received FNA or surgery provided informed consent prior to the procedures. The inclusion criteria were: (a) those who underwent conventional US, SMI, and FNA in our department between January 2020 and December 2021; (b) those with nodules > 5 mm in maximum diameter, as detected by routine US; (c) those with an interval not more than 2 weeks between FNA and surgery, during which time, they received no clinical intervention; and (d) those with pathologically confirmed benign or malignant nodules. Additionally, we eliminated the following patients from the analysis: (a) those with nodules stratified as Bethesda I, III, or IV based on the Bethesda Thyroid Cell Pathology Reporting System, with no evident pathological reports; (b) those with prior FNA or ablation; and (c) those with poor image quality, with severe artifacts or low resolution. The training and internal validation cohorts were recruited from the Second Affiliated Hospital of Zhejiang University School of Medicine, and the external validation cohort was obtained from the First Affiliated Hospital of Ningbo University.

### Pathological reference standards

The pathological reference standards were defined as follows: (a) surgical resection, as evidenced by histology; (b) the Bethesda system-based classification of malignant results (V, VI) without histopathological assessment; and (c) employment of the Bethesda system (II), for benign thyroid nodule identification without histopathological assessment, with the depiction of nodule stability or reduced size seen on the US over a 12-month follow-up period [24].

### US protocol

US-based assessments utilized a Canon Aplio i900 (Canon Medical System Corporation, Canon, Japan) equipped with an i18LX5 high-frequency (8–15 MHz) line array transducers, with conventional US and SMI functions. All participants were initially evaluated using conventional US, using parameters as follows: depth of 2.5–3 cm, with a focus on the middle to rear edge of the nodule, and dynamic range of 65 dB. Upon detection of a thyroid nodule, we recorded its size, position, and other US features, such as, composition, echogenicity, shape, margin, echogenic foci, halo, and extrathyroidal extension condition. Subsequently, we employed SMI to assess the vascular profile and morphology using parameters as follows: velocity scale, 1–1.5 cm/s; frame rate, 59 fps; and color gain, 41 dB. During the US procedure, patients were recommended to avoid swallowing and to breathe slowly to minimize motion. We also applied slight pressure through the transducer during imaging to avoid vessel collapse. Using the aforementioned process, we evaluated the vasculature in and around the lesions.

### Statistical analysis

We based our participant recruitment on 31% carcinoma incidence according to the ACR TI-RADS study, as well as an estimate of 85% sensitivity for the RSS for target lesions [25]. We attempted to include at least 561 thyroid nodules to yield a 91% power for 75–85% sensitivity detection using a two-sided binomial test. Continuous data were assessed via the unpaired *t*-test, whereas, categorical data utilized the χ^2^ test. Univariable logistic regression analysis was employed for the determination of significant US features (*p* value < 0.05), which were subsequently entered into the multivariable analysis. Thereafter, we rounded the average regression coefficients from multivariable analysis following 10-fold cross-validation to obtain scores for SMI TI-RADS generation, which were modeled after the ACR TI-RADS, using previously described methods. SMI TI-RADS, along with eight representative classification systems, namely, C TI-RADS, ACR TI-RADS, European Thyroid Association system (EU TI-RADS), Korean Society of Thyroid Radiology Imaging Guidelines (KSThR TI-RADS), American Association of Clinical Endocrinologists system (AACE TI-RADS), American Thyroid Association Management Guidelines (ATA TI-RADS), British Thyroid Association system (BTA TI-RADS), and French Society of Endocrinology system (FSE TI-RADS), were then employed for thyroid nodule evaluation. To elucidate each stratification system performance for patient biopsy requirement, we computed the biopsy yield of malignancy (BYM, representing the percentage of malignant nodules in the overall number of nodules required for biopsy) and the unnecessary biopsy rate (UBR, representing the percentage of benign nodules in the overall number of nodules required for biopsy), as described in a prior investigation, nodules with indications for biopsy were considered positive and those without indications for biopsy were considered negative. We next compared the SMI TI-RADS diagnostic and biopsy performances against the eight aforementioned classification systems.

Data analyses utilized the R-Project and R Studio (version 4.3.1), and two-tailed *p* < 0.05 was regarded as significant.

## Results

### Baseline demographics of study participants

**Fig. 1:**
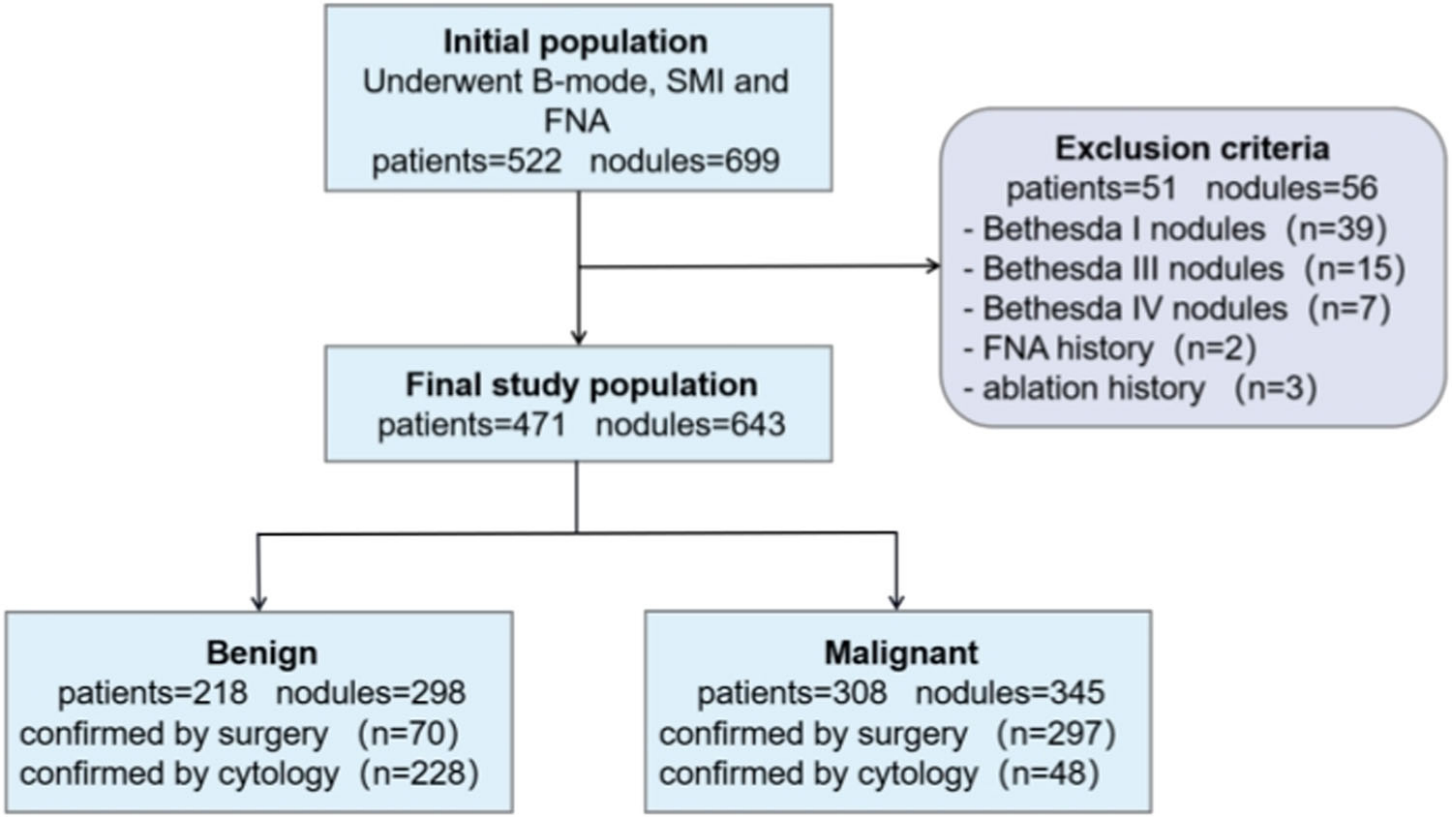
Flow diagram of the included patients and number of thyroid nodules. SMI, superb microvascular imaging; FNA, fine-needle aspiration; n, number of thyroid nodules

Table 1 details the baseline profiles of all patients. Our initial sample size included 522 patients with 699 nodules, recruited from patients who received conventional US, SMI mode US, and FNA at our institution between January 2020 and December 2021. Of these, 5 nodules were eliminated owing to a prior history of FNA (*n* = 2) or ablation (*n* = 3). Additionally, 56 nodules, particularly, 39 Bethesda I, 15 Bethesda III, and 7 Bethesda IV nodules, were eliminated due to unavailable pathological reference standards (Fig. 1). In the final training cohort, there were 471 participants (median age, 42 years [IQR, 34–54 years]), among which 103 were males (median age, 41 years [IQR, 34–53 years]) and 368 were females (median age, 42 years [IQR, 34–54 years]). Moreover, the total nodules were 643 (median size 10.9 mm, 7.5–13.9 mm), among which, 298 were benign thyroid nodules (median size 10.8 mm, [IQR, 7.2–15.8 mm]) and 345 were malignant thyroid nodules (median size 10.9 mm, [IQR, 7.9–12.8 mm]).

**Table 1:** Baseline data of training set and validation sets

Parameter	Training set	*p* value	Internal validation cohort	*p* value	External validation cohort	*p* value
Number of nodules	643		159		111	
Sex^&^						
F	510 (79.3)		133 (83.6)		86 (77.5)	
M	133 (20.7)		26 (16.4)		25 (22.5)	
Age (y)*		0.76		0.68		0.43
F	42 (34, 54)		43 (34, 56)		48.5 (36, 56)	
M	41 (34, 53)		40 (35, 48.5)		42 (34, 49)	
Nodules^&^						
Benign	298 (46.3)		58 (36.5)		50 (45.0)	
Malignant	345 (53.7)		101 (63.5)		61 (55.0)	
Size*		0.070		0.213		< 0.001
Benign	10.80 (7.23, 15.80)		10.90 (7.20, 16.58)		11.95 (10.22, 16.75)	
Malignant	10.90 (7.90, 12.80)		10.60 (7.40, 13.10)		10.20 (7.10, 12.40)	
Position^&^						
Left	287 (44.6)		75 (47.2)		49 (44.1)	
Right	320 (49.8)		74 (46.5)		55 (49.5)	
Isthmus	36 (5.6)		14 (8.8)		7 (6.3)	

^*^ Median with 95% CI

^&^ Data are numbers of nodules, with percentages in parentheses

Between the period of January 2022 and June 2022, 159 thyroid nodules from 144 patients (median age, 42 years [IQR, 34–56 years]) were selected from our internal validation cohort, among which, 120 were females (median age, 43 years, [IQR, 34–56 years]) and 24 were males (median age, 40 years, [IQR, 35–49 years]). Additionally, between January 2021 and December 2021, as our external validation cohort, we selected 111 thyroid nodules from 106 patients (median age, 46 years [IQR, 36–56 years], among which, 81 were females (median age, 49 years, [IQR, 36–56 years]) and 25 were males (median age, 42 years, [IQR, 34–49 years]). Table 1 summarizes the baseline profiles of internal and external validation cohorts.

### Development of the SMI TI-RADS estimation model

**Fig. 2:**
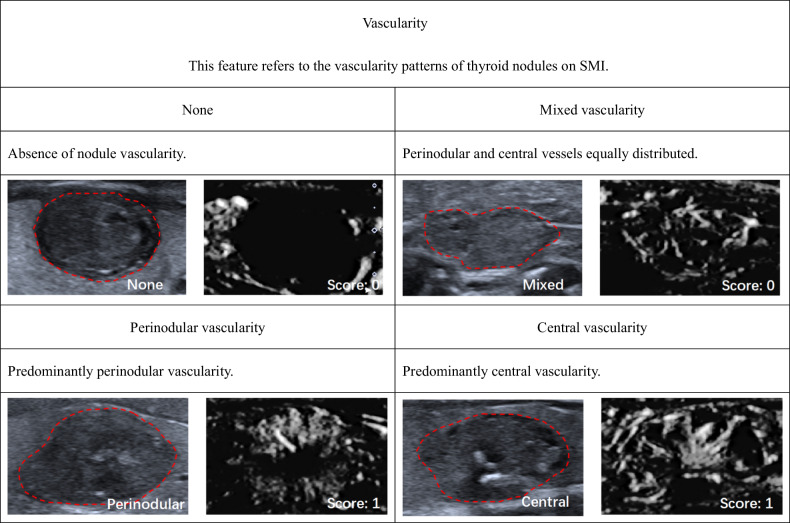
Monochrome SMI and conventional US images show features of vascularity

**Fig. 3:**
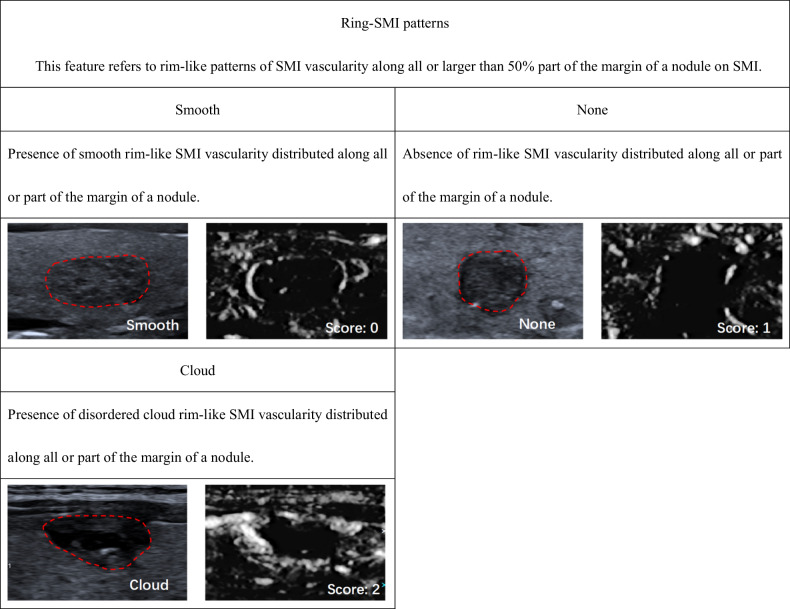
Monochrome SMI and conventional US images show features of ring-SMI patterns

**Fig. 4:**
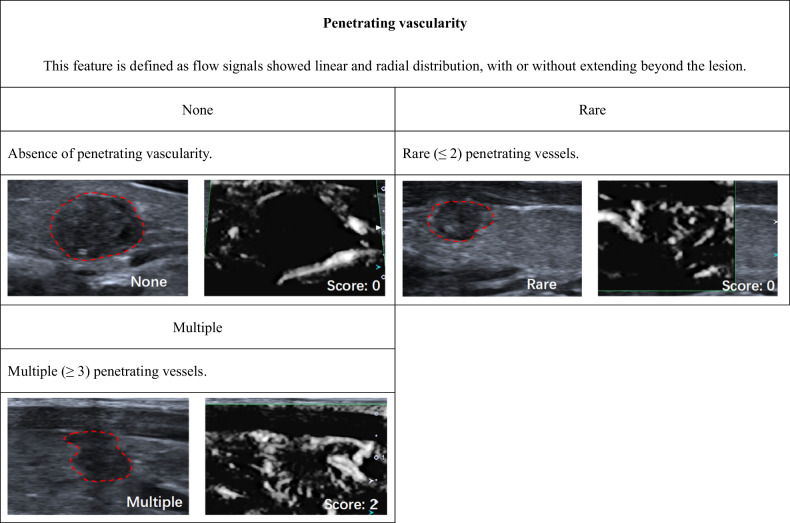
Monochrome SMI and conventional US images show features of penetrating vascularity

**Fig. 5:**
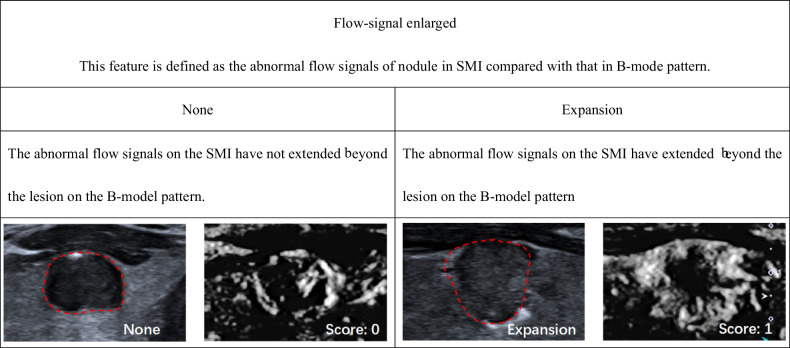
Monochrome SMI and conventional US images show features of the area enlarged

According to ACR TI-RADS, we established SMI TI-RADS for thyroid nodule malignancy prediction and FNA indication assessment. The univariable (Table S1 [online]) and multivariable logistic regression analyses indicated that nodule shape, margins, echogenicity, echogenic foci, extrathyroidal extension, vascularity, ring-SMI pattern, penetrating vascularity, flow-signal enlarged were strong indicators of thyroid carcinoma, and therefore, these parameters were employed for establishment of the SMI TI-RADS estimation model (Tables 2 and S2 [online]). Within this model, hyperechoic or isoechoic, shapes that were wider rather than tall, smooth margins, a lack of calcification or large comet tail artifacts, absence of an extrathyroidal extension, absent or mixed type of vascularity, smooth ring-SMI pattern, none too rare penetrating vascularity, and absent of flow-signal enlarged were given 0 points, while nodules that were either hypoechoic or highly hypoechoic, margins that were lobulated or irregular, peripheral or macrocalcifications, extrathyroidal extensions, central or perinodular type vascularity, absent of ring-SMI patterns, and present of flow-signal enlarged were given 1 point, and punctate echogenic foci, showing greater height than width, punctate echogenic foci, multiple penetrating vascularity, and cloud ring-SMI pattern were given 2 points (Figs. 2–5 and S1 [online]).

**Table 2:** Association between thyroid nodule malignancy and various SMI features

	Benign	Malignant	Univariable analysis		Multivariable analysis		
Features	(*n* = 298)	(*n* = 345)	β	*p* value^#^	β^&^	*p* value^#^	Score^▲^
Vascularity				< 0.001			
None/mixed	218	143	N/A		N/A	N/A	0
Perinodular/central	80	202	1.35		1.44 (1.38, 1.49)	< 0.001	1
Vascularity area ratio				0.475			
< 1/3	151	164	N/A		N/A	N/A	N/A
≥ 1/3	147	181	0.13		N/A	N/A	N/A
Ring-SMI patterns				< 0.001			
Smooth	216	115	N/A		N/A	N/A	0
None	44	51	0.78		1.21 (1.12, 1.29)	< 0.001	1
Cloud	38	179	2.18		2.30 (2.24, 2.36)	< 0.001	2
Penetrating vascularity				< 0.001			
None	249	135	N/A		N/A	N/A	0
Rare	26	19	0.30		0.53 (0.42, 0.63)	0.158	0
Multiple	23	191	2.73		2.40 (2.33, 2.47)	< 0.001	2
Flow-signal enlarged				< 0.001			
None	256	189	N/A		N/A	N/A	0
Expansion	42	156	1.62		1.31 (1.27, 1.35)	< 0.001	1

^#^ Determined with logistic regression analysis

^&^ Mean and 95% CI of regression coefficients of significant predictive both US and SMI features after 10-fold cross-validation

^▲^ Scoring criteria for significant independent predictors were based on the severely rounded mean of regression coefficients after 10-fold cross-validation to the nearest integer

**Fig. 6:**
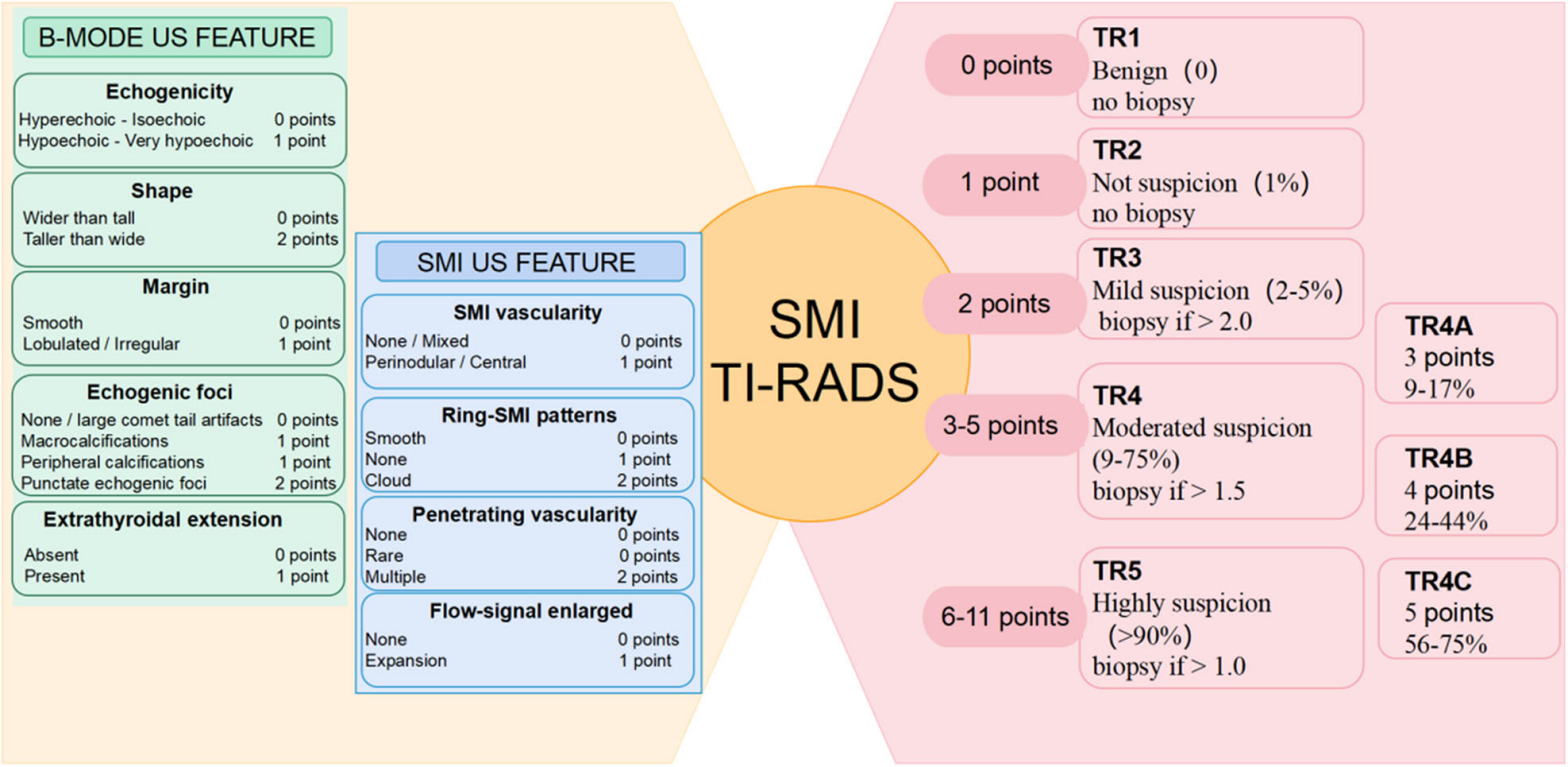
Chart shows the five categories of the SMI TI-RADS, with FP and indications for FNA

The total points determined the nodule SMI TI-RADS category (referred to as TR hereafter): 0 points represented TR1 (benign thyroid nodules; fitted probabilities [FP]: 0%), 1 point indicated TR2 (not suspicious; FP: 1%), 2 points meant TR3 (mildly suspicious; FP: 2–5%), 3 points represented TR4A (moderately suspicious; FP: 9–17%), 4 points indicated TR4B (moderately suspicious; FP: 24–44%), 5 points meant TR4C (moderately suspicious; FP: 56–75%), and 6–11 points represented TR5 (highly suspicious; FP: > 90%) (Fig. S2 [online]). The indication for FNA was dependent on an integration of nodule maximum diameter and classification (Fig. 6).

### Comparison between SMI TI-RADS and other risk classification systems

In all, we employed nine RSSs (namely, C TI-RADS, ACR TI-RADS, EU TI-RADS, KSThR TI-RADS, AACE TI-RADS, ATA TI-RADS, and SMI TI-RADS) to assess the risk of malignancy in the classifications of these guidelines. The application of the RSS was generally within the recommended range of malignancy, apart from the moderate and mild suspicion and not suspicious or benign classifications of ACR TI-RADS, the intermediate and low-risk classifications of EU TI-RADS, and the intermediate, low, and very low suspicion classifications of ATA TI-RADS. Beyond this, approximately 6.5% of the nodules (42/643) did not meet any of the classification standards in ATA TI-RADS, and showed a malignant risk of 57% (24 of 42 nodules). The other two RSSs (namely, BTA TI-RADS and FSE TI-RADS) did not propose malignancy risk estimates for each category (Table S3 [online]).

To test the efficiencies of the RSS diagnoses and evaluations, we compared the sensitivities, specificities, and positive and negative predictive values at an optimal diagnostic cut-off, as summarized in Table 3. The area under the receiver operating characteristic curve (AUC) of SMI TI-RADS (0.94 [95% CI: 0.92, 0.96]) was relatively enhanced, compared to that of C TI-RADS (0.86 [95% CI: 0.83, 0.89], *p* < 0.001), EU TI-RADS (0.84 [95% CI: 0.81, 0.86], *p* < 0.001), ACR TI-RADS (0.84 [95% CI: 0.81, 0.87], *p* < 0.001), KSThR TI-RADS (0.80 [95% CI: 0.77, 0.83], *p* < 0.001), AACE TI-RADS (0.86 [95% CI: 0.83, 0.88], *p* < 0.001), ATA TI-RADS (0.84 [95% CI: 0.81, 0.87], *p* < 0.001), FSE TI-RADS (0.82 [95% CI: 0.79, 0.85], *p* < 0.001), and BTA TI-RADS (0.76 [95% CI: 0.73, 0.80], *p* < 0.001) (Fig. S3 [online]). The SMI TI-RADS exhibited the largest malignancy biopsy yield at 79% (189/240 nodules), and was closely followed by ACR TI-RADS at 74% (190/258 nodules), AACE TI-RADS at 72% (197/273 nodules), FSE TI-RADS at 68% (191/280 nodules), EU TI-RADS at 66% (200/302 nodules), C TI-RADS at 64% (211/332 nodules), ATA TI-RADS at 61% (210/342 nodules), KSThR TI-RADS at 59% (211/355 nodules), and BTA TI-RADS at 59% (328/557 nodules) (Fig. S4 [online]).

**Table 3:** Comparison of the diagnostic performances of TI-RADS

Guideline	Sensitivity, (%)	Specificity, (%)	PPV, (%)	NPV, (%)	AUC	*p* value*
C TI-RADS	87 (84, 91) [301/345]	85 (81, 89) [252/298]	87 (83, 90) [301/347]	85 (81, 89) [252/296]	0.86 (0.83, 0.89)	< 0.001
ACR TI-RADS	82 (78, 86) [284/345]	86 (82, 90) [256/298]	87 (84, 91) [284/326]	81 (76, 85) [256/317]	0.84 (0.81, 0.87)	< 0.001
EU TI-RADS	89 (86, 93) [308/345]	78 (73, 83) [232/298]	82 (79, 86) [308/374]	86 (82, 90) [232/269]	0.84 (0.81, 0.86)	< 0.001
KSThR TI-RADS	84 (80, 88) [289/345]	77 (72, 81) [228/298]	81 (76, 85) [289/359]	80 (76, 85) [228/284]	0.80 (0.77, 0.83)	< 0.001
AACE TI-RADS	90 (87, 94) [312/345]	81 (76, 85) [241/298]	85 (81, 88) [312/369]	88 (84, 92) [241/274]	0.86 (0.83, 0.88)	< 0.001
ATA TI-RADS	85 (81, 89) [273/321]	82 (78, 87) [230/280]	85 (81, 89) [273/323]	83 (78, 87) [230/278]	0.84 (0.81, 0.87)	< 0.001
BTA TI-RADS	70 (65, 74) [240/345]	83 (79, 87) [247/298]	83 (78, 87) [240/291]	70 (65, 75) [247/352]	0.76 (0.73, 0.80)	< 0.001
FSE TI-RADS	89 (85, 92) [306/345]	75 (70, 80) [224/298]	81 (77, 85) [306/380]	85 (81, 90) [224/263]	0.82 (0.79, 0.85)	< 0.001
SMI TI-RADS	87 (84, 91) [301/345]	87 (83, 91) [259/298]	89 (85, 92) [301/340]	86 (82, 89) [259/303]	0.94 (0.92, 0.96)	NA

The data in parentheses represent a 95% CI value; the data in brackets represent the corresponding number and total number of the nodule

*C TI-RADS* Chinese Society of Ultrasound in Medicine system, *ACR TI-RADS* the American College of Radiology Thyroid Imaging Reporting and Data System, *EU TI-RADS* European Thyroid Association Guidelines for Ultrasound Malignancy Risk Stratification of Thyroid Nodules, *KSThR TI-RADS* Korean Society of Thyroid Radiology Imaging Guidelines for Thyroid Nodules and Differentiated Thyroid Cancer, *AACE TI-RADS* American Association of Clinical Endocrinologists Guidelines for Clinical Practice for the Diagnosis and Management of Thyroid Nodules, *ATA TI-RADS* American Thyroid Association Management Guidelines for Adult Patients with Thyroid Nodules and Differentiated Thyroid Cancer, *BTA TI-RADS* British Thyroid Association 2014 classification ultrasound scoring Guidelines of thyroid nodules in predicting malignancy, *FSE TI-RADS* Guidelines of the French Society of Endocrinology for the management of thyroid nodules, *PPV* positive predictive value, *NPV* negative predictive value, *AUC* area under the curve, *NA* not applicable

^*^ Compared the AUC between SMI TI-RADS and other TI-RADS systems

The SMI TI-RADS also displayed the least UBR at 21% (51/240 nodules), closely accompanied by ACR TI-RADS at 26% (68/258 nodules), AACE TI-RADS at 28% (76/273 nodules), FSE TI-RADS at 32% (89/280 nodules), EU TI-RADS at 34% (102/302 nodules), C TI-RADS at 36% (121/332 nodules), ATA TI-RADS at 39% (132/342 nodules), KSThR TI-RADS at 41% (144/355 nodules), and BTA TI-RADS at 41% (229/557 nodules). For the purposes of this study, nodules with indications for biopsy were considered positive and those without indications for biopsy were considered negative, their sensitivities, specificities, and positive and negative predictive values are presented in Table 4.

**Table 4:** Comparison of the biopsy performance of TI-RADS

Guideline	Sensitivity, (%)	Specificity, (%)	PPV, (%)	NPV, (%)	AUC	BYM, (%)	UBR, (%)
C TI-RADS	61 (56, 66) [211/345]	59 (54, 65) [177/298]	64 (58, 69) [211/332]	57 (51, 62) [177/311]	0.60 (0.56, 0.64)	64 (211/332)	36 (121/332)
ACR TI-RADS	55 (50, 60) [190/345]	77 (72, 82) [230/298]	74 (68, 79) [190/258]	60 (55, 65) [230/385]	0.66 (0.63, 0.70)	74 (190/258)	26 (68/258)
EU TI-RADS	58 (53, 63) [200/345]	66 (60, 71) [196/298]	66 (61, 72) [200/302]	58 (52, 63) [196/341]	0.62 (0.58, 0.66)	66 (200/302)	34 (102/302)
KSThR TI-RADS	61 (56, 66) [211/345]	52 (46, 57) [154/298]	59 (54, 65) [211/355]	54 (48, 59) [154/288]	0.56 (0.53, 0.60)	59 (211/355)	41 (144/355)
AACE TI-RADS	57 (52, 62) [197/345]	75 (70, 79) [222/298]	72 (67, 78) [197/273]	60 (55, 65) [222/370]	0.66 (0.62, 0.69)	72 (197/273)	28 (76/273)
ATA TI-RADS	61 (56, 66) [210/345]	56 (50, 61) [166/298]	61 (56, 67) [210/342]	55 (50, 61) [166/301]	0.58 (0.54, 0.62)	61 (210/342)	39 (132/342)
BTA TI-RADS	95 (93, 97) [328/345]	23 (18, 28) [69/298]	59 (55, 63) [328/557]	80 (72, 89) [69/86]	0.59 (0.56, 0.62)	59 (328/557)	41 (229/557)
FSE TI-RADS	55 (50, 61) [191/345]	70 (65, 75) [209/298]	68 (63, 74) [191/280]	58 (53, 63) [209/363]	0.63 (0.59, 0.66)	68 (191/280)	32 (89/280)
SMI TI-RADS	55 (50, 60) [189/345]	83 (79, 87) [247/298]	79 (74, 84) [189/240]	61 (57, 66) [247/403]	0.69 (0.65, 0.72)	79 (189/240)	21 (51/240)

*C TI-RADS* Chinese Society of Ultrasound in Medicine system, *ACR TI-RADS* The American College of Radiology Thyroid Imaging Reporting and Data System, *EU TI-RADS* European Thyroid Association Guidelines for Ultrasound Malignancy Risk Stratification of Thyroid Nodules, *KSThR TI-RADS* Korean Society of Thyroid Radiology Imaging Guidelines for Thyroid Nodules and Differentiated Thyroid Cancer, *AACE TI-RADS* American Association of Clinical Endocrinologists Guidelines for Clinical Practice for the Diagnosis and Management of Thyroid Nodules, *ATA TI-RADS* American Thyroid Association Management Guidelines for Adult Patients with Thyroid Nodules and Differentiated Thyroid Cancer, *BTA TI-RADS* British Thyroid Association 2014 classification ultrasound scoring Guidelines of thyroid nodules in predicting malignancy, *FSE TI-RADS* Guidelines of the French Society of Endocrinology for the management of thyroid nodules, *PPV* positive predictive value, *NPV* negative predictive value, *AUC* area under the curve, *BYM* biopsy yield of malignancy, *UBR* unnecessary biopsy rate

### Validation

In the internal validation cohort, the AUC, sensitivity, specificity, BYM, and UBR of the SMI TI-RADS were 0.89 (95% CI: 0.83, 0.94), 0.83 (95% CI: 0.76,0.91), 0.85 (95% CI: 0.75, 0.94), 79% (59/75 nodules), and 21% (16/75 nodules), respectively. In the external validation cohort, the AUC, sensitivity, specificity, BYM, and UBR of the SMI TI-RADS were 0.91 (95% CI: 0.85, 0.96), 0.89 (95% CI: 0.81, 0.97), 0.86 (95% CI: 0.76, 0.96), 72% (31/43 nodules), and 28% (12/43 nodules), respectively.

### Evaluation of interobserver variability

Based on our interobserver variability assessment, the κ value was 0.63 for echogenicity, 0.72 for shape, 0.75 for margin, 0.52 for echogenic foci, 0.66 for extrathyroidal extension, 0.46 for vascularity at SMI mode, 0.55 for ring-SMI patterns, 0.70 for penetrating vascularity, and 0.60 for flow-signal enlarged at SMI, respectively (Table S4 [online]).

## Discussion

To explore the significance of vascular characteristics in the stratification of malignancy risk in thyroid nodules, we redefined SMI features and combined them with conventional US features to establish the SMI TI-RADS. All features in our hierarchical system were qualitative features, and this point-based approach allowed all thyroid nodules to be classified, ensuring the practicality of this stratification system.

Malignant tumors are characterized by abnormal vascular distribution and the presence of irregular vasculature [26–30]. However, due to limitations in current imaging methods and use, they had not yet been included in the TI-RADS stratification system. In recent years, in response to the shortcomings of conventional US and CDFI imaging, imaging techniques capable of assessing low levels of blood flow and smaller blood vessels have been developed [31–34]. These have provided more detailed images of microvascular branches and the distribution of blood flow in both the nodule and the adjoining parenchyma, providing an accurate evidence-based foundation for the stratification of malignancy risk in thyroid nodules to better guide clinical diagnosis and treatment.

We thus constructed a novel system, using the addition of SMI features to predict thyroid nodule malignancy by the rounded regression coefficient, SMI TI-RADS, covering features such as SMI vascularity, ring-SMI patterns, penetrating vascularity, and flow-signal enlargement. We also provided a detailed explanation of the above features to increase the practicality of our system, and scored thyroid nodules directly to obtain the risk stratification. It was found that the AUC, sensitivity, and specificity of the SMI TI-RADS were 0.94 (95% CI: 0.92, 0.96), 0.87 (95% CI: 0.84, 0.91), and 0.87 (95% CI: 0.83, 0.91), respectively.

There is no clear consensus on the requirements for needle biopsy among the different TI-RADS stratification systems. Without biopsy, approximately 26.8‒27.7% of patients with thyroid nodules will need to undergo active surveillance for a long time, leading to a certain amount of psychological pressure in the patients [9]. Therefore, it is important to consider the size threshold for biopsy when developing TI-RADS risk stratification, seeking high specificity and minimizing unnecessary biopsies [35, 36]. Compared with the remaining eight RSSs, our results showed that the specificity, BYM, and UBR of SMI TI-RADS were 0.83 (95% CI: 0.79, 0.87), 79%, and 21%, respectively.

Our study has several limitations. First, the study was retrospective and selection bias is thus inevitable. As our institution is a Class A tertiary hospital, the majority of patients were likely referred from local centers or clinics after the discovery of suspicious findings. Furthermore, we only included patients with thyroid nodules who had received SMI- and US-guided FNA or surgical resection, which may have resulted in a higher likelihood of nodule malignancy. Second, owing to the principle of Doppler US imaging-based SMI examination, the blood flow display was predictably impacted by the nodule location depth and the cardiac cycle, which caused certain biases in the selection of data. Third, we did not include multicenter data. The accuracy of the SMI TI-RADS stratification system we constructed thus requires further verification using more imaging data, including multi-center training cohorts and validation cohorts. Fourth, the interpretation of all the indicators used for evaluation, such as conventional US, SMI features, and FNA results, was dependent on the doctor’s level of experience and may thus be subject to inter-observer differences.

In summary, we redefined SMI-related features and classification criteria, and successfully constructed the SMI TI-RADS stratification system based on ACR TI-RADS. We recommend additional prospective multicenter verification of our RSS prior to widespread clinical application.

## Supplementary information

ELECTRONIC SUPPLEMENTARY MATERIAL

## Data Availability

The datasets analyzed for the current study are not publicly available due to patient privacy. Reasonable requests will be reviewed individually for application to the corresponding author.

## Abbreviations

AUC: Area under the curve
BYM: Biopsy yield of malignancy
FNA: Fine-needle aspiration
MVUS: Microvascular ultrasound
SMI: Superb microvascular imaging
TI-RADS: Thyroid imaging reporting and data system
UBR: Unnecessary biopsy rate
US: Ultrasound
