# Supplementary material for: Application of microvascular ultrasound-assisted thyroid imaging report and data system in thyroid nodule risk stratification
Source: Insights Imaging. 2024 Sep 23;15:230. doi: 10.1186/s13244-024-01806-5 (PMC11420409; doi:10.1186/s13244-024-01806-5)
Supplement: Supplementary file 1 — ELECTRONIC SUPPLEMENTARY MATERIAL [file 13244_2024_1806_MOESM1_ESM.pdf]

Application of microvascular ultrasound-assisted thyroid imaging report and data system in thyroid nodule risk stratification

ELECTRONIC SUPPLEMENTARY MATERIAL

US Image Analysis

The B-mode and the monochrome superb microvascular imaging (SMI) mode ultrasound images of the thyroid nodules were independently analyzed by 2 senior radiologists with more than ten-years-experience in clinical working who blinded to the histology pathology results. If there are differences of opinion, invite a third-party radiologist with similar experience to participate in the final decision.

The B-mode ultrasound features we interested in including: composition of the nodules (nonsolid or solid, nonsolid nodules includes mixed solid and cystic nodules, spongiform nodules, and cysts); echogenicity of nodules (hyperechoic or isoechoic relative to adjacent thyroid tissue, hypoechoic relative to adjacent thyroid tissue, very hypoechoic relative to anterior neck muscles); shape of nodules (wider-than-tall or taller-than-wide); margin of nodules (smooth, lobulated or irregular); echogenic foci of nodules (none, large comet tail artifacts, peripheral, macrocalcifications or punctate echogenic foci); extrathyroidal extension (absent or present). The monochrome superb microvascular imaging (SMI) mode ultrasound features we interested in including: vascularity of nodules in SMI mode (none; perinodular vascularity; central vascularity or mixed vascularity); vascularity area ratio of nodules in SMI mode (<1/3 or ≥1/3); ring-SMI patterns of nodules in SMI mode (smooth, none or cloud); penetrating vascularity number of nodules in SMI mode (none, rare or multiple); nodule flow-signal enlarged in SMI compared with that in B-mode pattern (none or expansion relative to nodules).

Evaluation of interobserver variability

We randomly selected the same fifty patients from the internal training set and had two radiologists with 5-10 years of clinical experience interpret indications in both B mode and SMI mode in a double-blind manner, and conducted a Kappa consistency test on the results. The classification criteria for consistency test results are as follows: no agreement equaling to 0.0, slight agreement ranging from 0.01 to 0.20, fair agreement ranging from 0.21 to 0.40, moderate agreement ranging from 0.41 to 0.60, substantial agreement ranging from 0.61 to 0.80, and almost excellent agreement ranging from 0.81 to 0.99, perfect agreement equaling to 1.0.

US Features and Their Definitions for Thyroid Nodules

Composition

Proportion of solid or cystic components in a nodule confirmed by nonenhanced US.

Solid.—

Composed entirely or nearly entirely of soft tissue, with no more than approximately 5% tiny cystic components. Nodules with shadowing calcifications that preclude evaluation of their architecture are assumed to be solid.

Mixed solid and cystic.—

Nodules that contain more than minimal solid components. There is no need to distinguish between predominantly solid and predominantly cystic nodules.

Spongiform.—

Nodules in which at least 50% of their volume is occupied by tiny cystic spaces. Nodules with other features, for instance, peripheral calcifications or macrocalcifications should not be classified as spongiform.

Cystic.—

Nodules composed entirely or nearly entirely of liquid.

Echogenicity

Echogenicity is used to describe the echo of solid components of a nodule compared with adjacent thyroid tissue, even in the case of diffuse thyroid disease. There is only one exception for very hypoechoic, in which anterior neck muscles are used as the object of reference.

Hyperechoic-Isoechoic.—

Nodule’s echogenicity is strong or similar to adjacent thyroid tissue. If dense calcification completely obstructs the reflectivity of a solid nodule, it is defined as hyperechoic.

Hypoechoic.—

Nodule’s echogenicity is below to adjacent thyroid tissue.

Very hypoechoic.—

Nodule’s echogenicity is below to anterior neck muscles.

Shape

The shape of thyroid nodule is evaluated in the axial plane by comparing the tallness (anteroposterior diameter measured by perpendicular to the ultrasound beam) and the width (left and right diameter measured by parallel to the ultrasound beam).

Wider than tall.—

This feature is defined as a ratio of ≤ 1 by contrast the tallness diameter to the horizontal diameter of a nodule in the axial plane, respectively

Taller than wide.—

This feather is defined as a ratio of > 1 by contrast the height (tallness) diameter to the width diameter of a nodule (measured perpendicular and parallel to the ultrasound beam) in the axial plane, respectively.

Margin

The feature of margin is evaluated by the boundary between the nodule and the adjacent thyroid tissue.

Smooth.—

Nodules with complete and clear boundary, or curvilinear edges without interruption.

Irregular and lobulated.—

Nodules with any protrusion of surface including needle shape、zigzag、acute angle and bow-shape with or without clear soft tissue protrusions into the parenchyma. The protrusion or lobulation may vary in size and quantity.

Echogenic Foci

Echogenic Foci refers to local markedly increased echogenicity compared to the surrounding tissue. This phenomenon also named calcification. Calcification may associate with several posterior acoustic artifacts according to its size and shape.

No calcification.—

Nodules with no calcification.

Large comet-tail artifacts.—

The calcification' size in nodule is so small which can produce a strong echogenic band more than 1 mm in depth.

Macrocalcification.—

The calcifications' size in nodules is larger than 1mm which can produce acoustic shadowing.

Peripheral calcification.—

The calcification locates at the edge of nodules.

Punctate echogenic foci.—

The calcifications' size in nodules is less than 1mm that can't produce shadowing. This category includes psammomatous calcifications.

Extrathyroidal Extension

Nodule grows out of the thyroid border.

Absent.—

Nodules locate within the thyroid, no extending through the thyroid gland.

Present.—

Nodules that extend throughout of the thyroid capsule.

Halo

Hypoechoic halo outside the nodule.

Absent.—

There is no hypoechoic halo outside the nodule.

Present.—

There is a hypoechoic halo outside the nodule.

Table S1 Coding of Ultrasound Features in The Logistic Regression Analysis

Variables	0	1	2
Composition*	Nonsolid	Solid	
Echogenicity	Hyperechoic/isoechoic	Hypoechoic/Very hypoechoic	
Shape	Wider than tall	Taller than wide	
Margin	Smooth	Lobulated/ Irregular	
Echogenic foci	None or large comet tail artifacts	Peripheral/Macrocalcifications	Punctate echogenic foci
Extrathyroidal extension	Absent	Present	
Halo	Absent	Present	
Vascularity	None/Mixed	Perinodular/Central	
Ring-SMI patterns	Smooth	None	Cloud
Penetrating vascularity	None	Rare	Multiple
Flow-signal enlarged	None	Expansion	
Vascularity area ratio	< 1/3	≥ 1/3	

*Nonsolid nodules included mixed solid and cystic nodules, spongiform nodules, and cysts.

Table S2 Association between Thyroid Malignancy and Various conventional US Features

	Benign	Malignant	Univariable Analysis		Multivariable Analysis		
Features	(n=298)	(n=345)	β	*P* Value[#]	β[&]	*P* Value[#]	Score[▲]
B-mode Features							
Composition				0.001			
Nonsolid	67	41	N/A		N/A	N/A	N/A
Solid	231	304	0.77		N/A	N/A	N/A
Echogenicity				< 0.001			
Hyperechoic / isoechoic	144	52	N/A		N/A	N/A	0
Hypoechoic / Very hypoechoic	154	293	1.66		1.32 (1.26,1.38)	< 0.001	1
Shape				< 0.001			
Wider than tall	270	205	N/A		N/A	N/A	0
Taller than wide	28	140	1.88		1.83 (1.75,1.90)	< 0.001	2
Margin				< 0.001			

Smooth	198	116	N/A		N/A	N/A	0
Lobulated or irregular	100	229	1.36		1.21 (1.16,1.26)	< 0.001	1
Echogenic Foci				< 0.001			
None or large comet tail artifacts	209	141	N/A		N/A	N/A	0
Peripheral/Macrocalcifications	33	61	1.01		0.88 (0.81,0.95)	0.003	1
Punctate echogenic foci	56	143	1.33		1.66 (1.60,1.71)	< 0.001	2
Extrathyroidal extension				< 0.001			
Absent	263	236	N/A		N/A	N/A	0
Present	35	109	1.24		1.28 (1.20,1.36)	< 0.001	1
Halo				0.001			
Absent	236	306	N/A		N/A	N/A	N/A
Present	62	39	-0.72		N/A	N/A	N/A

#: Determined with logistic regression analysis.
&: Mean and 95% CI of regression coefficients of significant predictive both US and SMI features after 10-fold cross-validation.
▲: Scoring criteria for significant independent predictors were based on the severely rounded mean of regression coefficients after 10-fold cross-validation to the nearest integer.

Table S3 Comparison of the Malignancy Risk of Different Thyroid Imaging Reporting and Data Systems

Guidelines and Categories	Final diagnosis		Recommended malignancy risk (%)	Calculated malignancy risk (%)	*P* Value*
	Benign (n=298)	Malignant (n=345)			
C TI-RADS					<0.001
High suggestion	0 (0.0)	4 (1.2)	> 90	100	
High suspicion	46 (15.4)	297 (86.1)	50-90	87	
Moderate suspicion	89 (29.9)	36 (10.4)	10-50	29	
Low suspicion	102 (34.2)	7 (2.0)	2-10	6	
Probably benign	59 (19.8)	1 (0.3)	<2	2	
Benign	2 (0.7)	0 (0.0)	0	0	
ACR TI-RADS					<0.001
High suspicion	42 (14.1)	284 (82.3)	>20	87	
Moderate suspicion	75 (25.2)	49 (14.2)	5-20	40	
Mild suspicion	138 (46.3)	9 (2.6)	<2	6	
Not suspicion/Benign	43 (14.4)	3 (0.9)	<2	7	
EU TI-RADS					<0.001
High risk	66 (22.1)	308 (89.3)	26-87	82	
Intermediate risk	63 (21.1)	28 (8.1)	6-17	31	
Low risk	166 (55.7)	9 (2.6)	2-4	5	
Benign	3 (1.0)	0 (0.0)	0	0	
KSThR TI-RADS					<0.001

High suspicion	70 (23.5)	289 (83.8)	>60	81	
Intermediate suspicion	90 (30.2)	42 (12.2)	15-50	32	
Low suspicion	134 (45.0)	14 (4.1)	3-15	9	
Benign	4 (1.3)	0 (0.0)	<3	0	
AACE TI-RADS					<0.001
High risk	57 (19.1)	312 (90.4)	50-90	85	
Intermediate risk	238 (79.9)	33 (9.6)	5-15	12	
Low risk	3 (1.0)	0 (0.0)	1	0	
ATA TI-RADS					<0.001
High suspicion	50 (16.8)	273 (79.1)	>70-90	85	
Intermediate suspicion	71 (23.8)	26 (7.5)	10-20	27	
Low suspicion	131 (44.0)	21 (6.1)	5-10	14	
Very low suspicion	24 (8.1)	1 (0.3)	<3	4	
Benign	4 (1.3)	0 (0.0)	<1	0	
Unclassified	18 (6.0)	24 (7.0)	Not mentioned		
BTA TI-RADS					<0.001
Malignant	51(17.1)	240 (69.6)		82	
Suspicion	127(42.6)	52 (15.1)		29	
Intermediate	51 (17.1)	36 (10.4)		41	
Benign	69 (23.2)	17 (4.9)		20	
FSE TI-RADS					<0.001
≥2 suspicious features	74 (24.8)	306 (88.7)		81	

<2 suspicious features	224 (75.2)	39 (11.3)		15	
SMI TI-RADS					N/A
High suspicion	12 (4.0)	261 (75.7)	>90	96	
Moderated suspicion (4C)	27 (9.1)	42 (12.2)	57-75	61	
Moderated suspicion (4B)	43 (14.4)	30 (8.7)	25-45	41	
Moderated suspicion (4A)	65 (21.8)	7 (2.0)	9-17	10	
Mild suspicion	75 (25.2)	4 (1.2)	3-6	5	
Not suspicion	54 (18.1)	1 (0.3)	<2	2	
Benign	22 (7.4)	0 (0.0)	0	0	

* Determined with $\chi 2$ test;
C TI-RADS = Chinese Society of Ultrasound in Medicine system, ACR TI-RADS = The American College of Radiology Thyroid Imaging Reporting and Data System, EU TI-RADS = European Thyroid Association Guidelines for Ultrasound Malignancy Risk Stratification of Thyroid Nodules, KSThR TI-RADS = Korean Society of Thyroid Radiology Imaging Guidelines for Thyroid Nodules and Differentiated Thyroid Cancer, AACE TI-RADS = American Association of Clinical Endocrinologists Guidelines for Clinical Practice for the Diagnosis and Management of Thyroid Nodules, ATA TI-RADS = American Thyroid Association Management Guidelines for Adult Patients with Thyroid Nodules and Differentiated Thyroid Cancer, BTA TI-RADS = British Thyroid Association 2014 classification ultrasound scoring Guidelines of thyroid nodules in predicting malignancy, FSE TI-RADS = Guidelines of the French society of endocrinology for the management of thyroid nodules, AUC = area under the curve, NA = not applicable.

Table S4 Interobserver Variability of Predictive US Features

Features	Kappa	*P* Value	Observers (n)	Nodules (n)
Echogenicity	0.63	<0.001	2	50
Shape	0.72	<0.001	2	50
Margin	0.75	<0.001	2	50
Echogenic foci	0.52	<0.001	2	50
Extrathyroidal extension	0.66	<0.001	2	50
Vascularity	0.46	0.001	2	50
Ring-SMI patterns	0.55	<0.001	2	50
Penetrating vascularity	0.70	<0.001	2	50
Flow-signal enlarged	0.60	<0.001	2	50

Supplemental Information:

<table>
<tr><td colspan="2">Vascularity area ratio
This feature is defined as the ratio of nodule vascularity area on SMI.</td></tr>
<tr><td>Less than 1/3</td><td>No less than 1/3</td></tr>
<tr><td>Ratio of nodule vascularity area is less than 1/3 on SMI.</td><td>Ratio of nodule vascularity area is more than or equal to 1/3 on SMI.</td></tr>
<tr><td>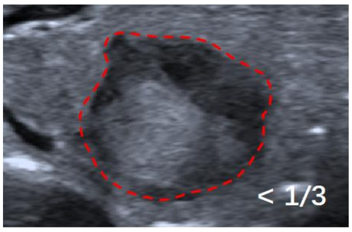

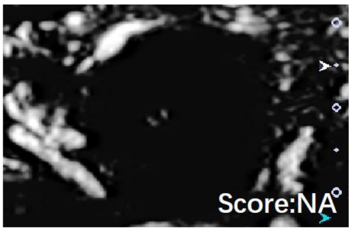
</td><td>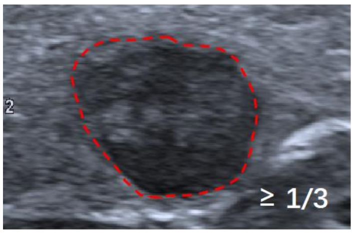

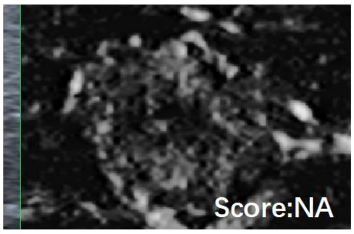
</td></tr>
</table>

Figure S1: Monochrome superb microvascular imaging (SMI) and conventional US images show features of vascularity area ratio. (NA = not applicable)

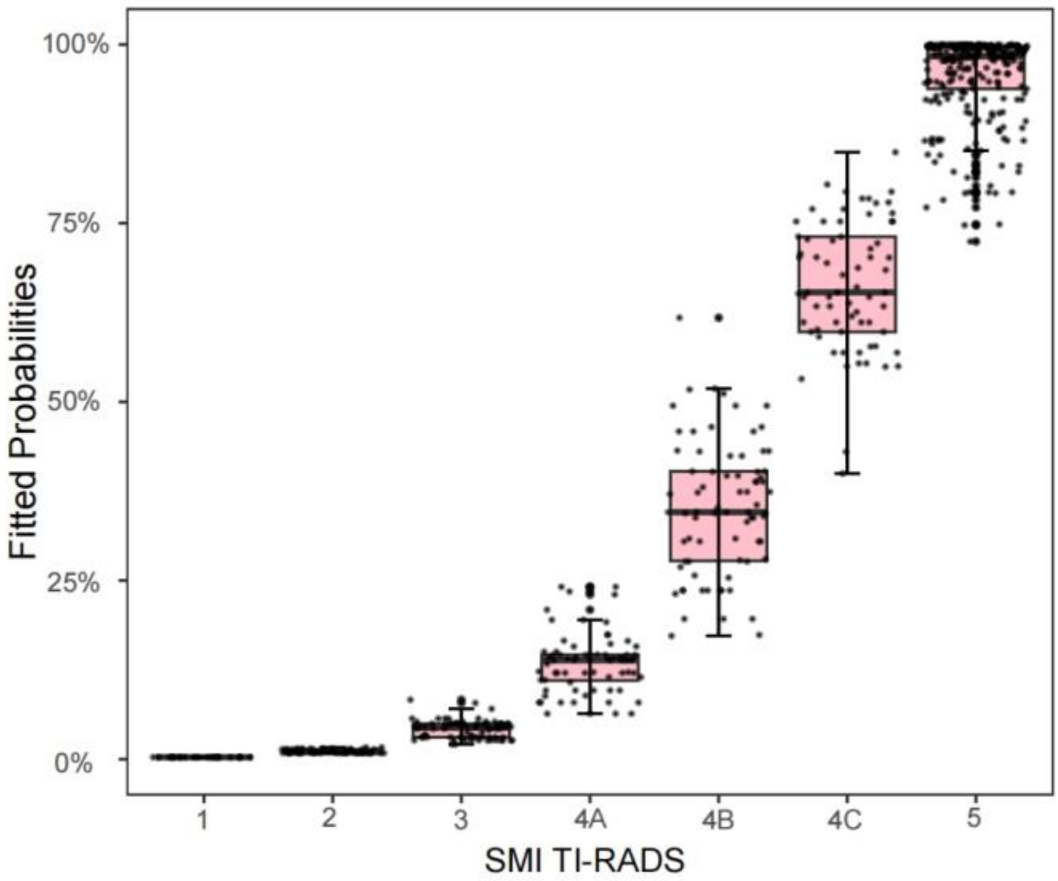

Figure S2: Box plot shows fitted probabilities of malignancy calculated with logistic regression by using US features of the 643 thyroid nodules. The lower and upper ends of vertical lines are minimum and maximum values of the malignant risk. The upper edge of the box is the 75th percentile of the data set; lower hinge represents the 25th percentile. The line in the box represents the median.

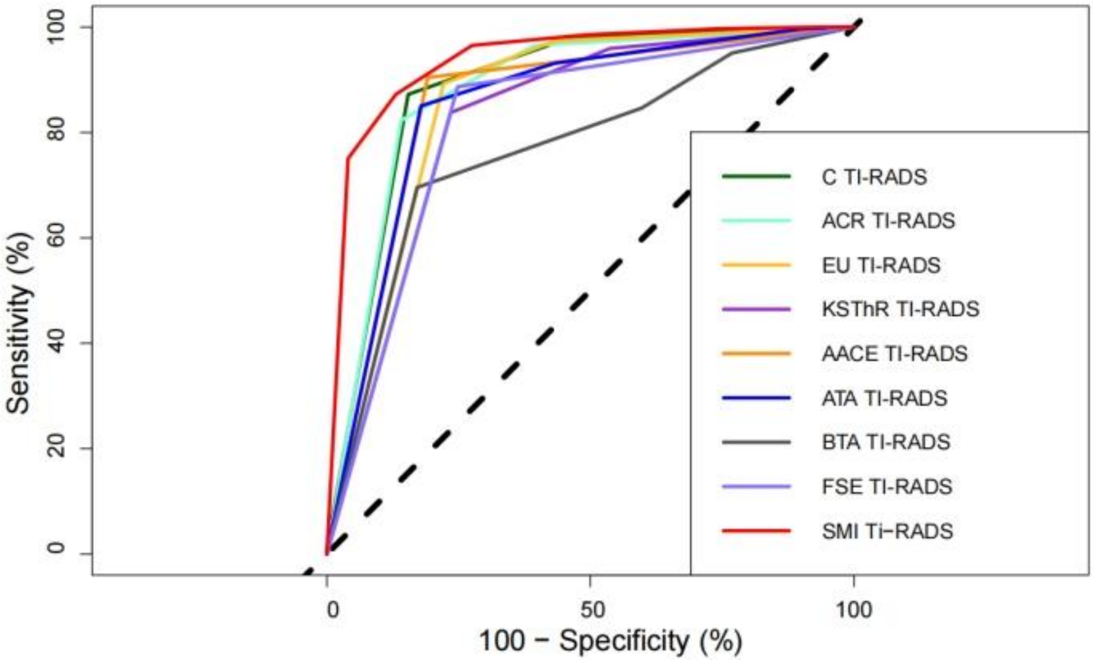

Figure S3: Receiver operator characteristic curves of different stratification systems when considering the final diagnosis of included nodules as the endpoint. C TI-RADS = Chinese Society of Ultrasound in Medicine system, ACR TI-RADS = The American College of Radiology Thyroid Imaging Reporting and Data System, EU TI-RADS = European Thyroid Association Guidelines for Ultrasound Malignancy Risk Stratification of Thyroid Nodules, KSThR TI-RADS = Korean Society of Thyroid Radiology Imaging Guidelines for Thyroid Nodules and Differentiated Thyroid Cancer, AACE TI-RADS = American Association of Clinical Endocrinologists Guidelines for Clinical Practice for the Diagnosis and Management of Thyroid Nodules, ATA TI-RADS = American Thyroid Association Management Guidelines for Adult Patients with Thyroid Nodules and Differentiated Thyroid Cancer, BTA TI-RADS = British Thyroid Association 2014 classification ultrasound scoring Guidelines of thyroid nodules in predicting malignancy, FSE TI-RADS = Guidelines of the French society of endocrinology for the management of thyroid nodules.

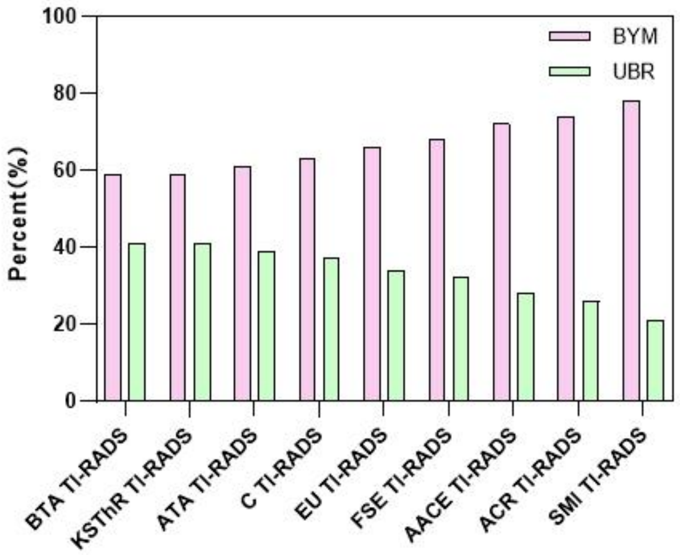

Figure S4: Biopsy yield of malignancy and unnecessary biopsy rate of different TI-RADS stratification systems. C TI-RADS = Chinese Society of Ultrasound in Medicine system, ACR TI-RADS = The American College of Radiology Thyroid Imaging Reporting and Data System, KSThR TI-RADS = Korean Society of Thyroid Radiology Imaging Guidelines for Thyroid Nodules and Differentiated Thyroid Cancer, American Association of Clinical Endocrinologists Guidelines for Clinical Practice for the Diagnosis and Management of Thyroid Nodules, ATA TI-RADS = American Thyroid Association Management Guidelines for Adult Patients with Thyroid Nodules and Differentiated Thyroid Cancer, FSE TI-RADS = Guidelines of the French society of endocrinology for the management of thyroid nodules.
